# Electrically Tunable Fresnel Lens in Twisted-Nematic Liquid Crystals Fabricated by a Sagnac Interferometer

**DOI:** 10.3390/polym11091448

**Published:** 2019-09-04

**Authors:** Bing-Yau Huang, Tsung-Hsien Lin, Tian-Yi Jhuang, Chie-Tong Kuo

**Affiliations:** 1 Department of Physics, National Sun Yat-sen University, Kaohsiung 804, Taiwan; 2 Department of Photonics, National Sun Yat-sen University, Kaohsiung 804, Taiwan; 3 Department of Optometry, Shu-Zen Junior College of Medicine and Management, Kaohsiung 821, Taiwan; 4 Innovation Incubation Center, Shu-Zen Junior College of Medicine and Management, Kaohsiung 821, Taiwan

**Keywords:** Fresnel lens, twisted nematic liquid crystal, Sagnac interferometer, azo dye

## Abstract

This paper presents an electrically tunable Fresnel lens in a twisted nematic liquid crystal cell fabricated by using a Sagnac interferometer. When the Fresnel-patterned green beam, formed by the Sagnac interferometer, is irradiated on the azo-dye doped liquid crystal mixture, the azo-dye molecules undergo *trans*–*cis* photoisomerization and then generate the photo-alignment effect in the bright (odd) zones. The director of the liquid crystal molecules in the odd zones reorients the direction perpendicular to the polarization direction of the linearly polarized green beam. The various structures of liquid crystals in the odd and even zones will result in a phase difference and thus, a Fresnel lens can be generated. The experimental results show that the proposed Fresnel lens has a high diffraction efficiency of 31.5% under an applied alternating-currents (AC) voltage. The focal length of the Fresnel lens can also be tuned by thermally erasing the photo-alignment effect of the azo dyes and rewriting by a different Fresnel-like pattern.

## 1. Introduction

Fresnel lenses are of enormous interest due to their wide range of applications [1,2,3,4,5,6,7,8,9,10]. Traditional Fresnel lenses are fabricated by thin-film deposition and electron-beam lithography [11,12], which leads to some disadvantages, such as the diffraction (focusing) efficiency being fixed and not being adjustable. Such issues can be overcome by using liquid crystals (LCs), which can be controlled by an electrical field.

The diffraction efficiency of LC Fresnel lens can be controlled by varying electrical fields because the orientations as well as the refractive indices of LCs in the odd and even zones can be modulated, resulting in the phase difference of light. In previous works, many methods for fabricating LC Fresnel lens have been proposed, such as using photomasking [13] and patterned alignment of LCs [14]. In order to achieve the various alignment directions for the LCs between the adjacent zones, many works focus on non-contact photoalignment technology [15], which can easily generate complex alignment structures. The methods of photomasking [13], Fresnel structure [16,17], and holographic techniques [18,19] are used to fabricate a Fresnel lens device. However, the common disadvantage of the above-mentioned LC Fresnel lenses is the fixed focal length. 

In this paper, Fresnel lenses in twisted nematic liquid crystals (TN-LCs) with different focal lengths can be achieved by using an interference method, which can be adjusted for the formation of a Fresnel-like pattern with various sizes. Fresnel lenses fabricated with twisted nematic liquid crystal configuration is expected to exhibit polarization insensitivity or even independence with the incident light when the external voltage is applied [20,21]. By irradiating the Fresnel-like pattern from a Sagnac interferometer on the sample, an electrically tunable Fresnel lens can be obtained. The diffraction efficiency of the LC Fresnel lens can be measured and modulated by applying alternating-currents (AC) voltage. In addition, the focal length can be varied by changing the position of the LC sample in the Sagnac interferometer.

## 2. Experiments

The azo dye-doped LCs (ADDLCs) in this experiment are prepared by doping the azo dyes, methyl red (MR, from Sigma-Aldrich, St. Louis, MO, USA), with a concentration of 0.5–2.0 wt%, into the LCs, E7 (*n_e_* = 1.603, *n_o_* = 1.486, from Merck (Darmstadt, Germany)). The mixture of the ADDLCs is injected into the empty cell, which is produced with two planar indium-tin oxide (ITO) glass plates separated by plastic spacers of 12 μm. Two ITO glass substrates are spin-coated with polyimide (PI, from Daily) films. One of the PI-coated ITO glass substrates is rubbed homogeneously to align the ADDLCs while the other one serves as a control substrate for the LC Fresnel lens. The ADDLC sample is further used for fabricating the LC Fresnel lens. The azo dyes exhibit two isomers, *trans*- and *cis*-isomers. The absorption band of the *trans*-isomer is about 400–575 nm. The maximum absorption is about 500 nm, while absorption is negligible above 600 nm. Therefore, in this experiment, a green laser with a wavelength of 532 nm is used as the pump beam, and a red laser of 632.8 nm is employed as the probe beam.

**Figure 1:**
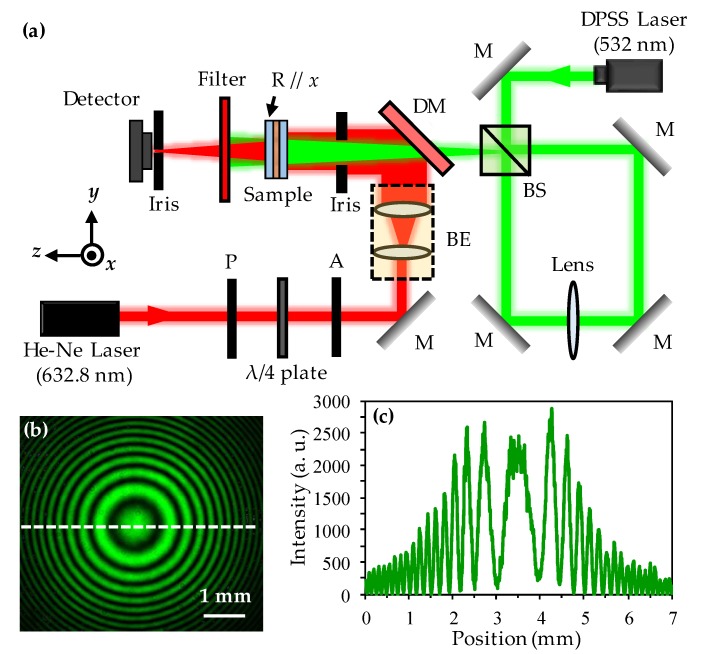
(**a**) Schematic illustration of the experimental setup for fabricating the Fresnel-like pattern in the azo dye-doped liquid crystal (ADDLC) sample and analyzing the focusing characteristics, where P is polarizer, A is analyzer, BE is beam expender, DM is dichroic mirror, BS is beam splitter, M is mirror, and R is the direction of rubbing alignment, respectively; (**b**) the Fresnel-like pattern created by the Sagnac interferometer; and (**c**) the cross-section of the intensity modulated with the position in the dashed line of (**b**).

Figure 1a schematically illustrates the experimental setup for fabricating the Fresnel-like pattern in the ADDLC sample and analyzing the focusing characteristics. The diode-pumped solid-state (DPSS) laser with a wavelength of 532 nm is incident on the beam splitter (BS) and is divided into two beams with equal intensities, which are aligned in the path of a Sagnac interferometer loop. Two beams propagating in the same path but along opposite directions in the loop will superpose and generate the Fresnel-like pattern [22]. The polarization of the green beam is parallel to the *x*-axis. Therefore, a spatial distribution of twist-nematic structures can be induced when the interference pattern illuminates on the controlled substrate of the ADDLC sample. A lens placed in the loop is used to magnify the Fresnel-like pattern. A He-Ne laser of 632.8 nm with linear polarization is used as the probe beam to measure the focusing properties of the ADDLC sample (rubbing direction parallel to *x*-axis), which are analyzed using a detector. The Fresnel-like pattern created by the Sagnac interferometer is shown in Figure 1b. The brightness in the center of the pattern is greater than that on the edges because the intensity distribution of the green beam is a Gaussian distribution. The cross-section of the modulated intensity along the direction of the dashed line of Figure 1b is shown in Figure 1c. The center radius of the Fresnel-like pattern is about 500 μm. The focal length (*f*), based on the theory of a binary phase Fresnel lens, can be written as:

(1) $$f=\frac{r_{1}^{2}}{\lambda },$$

where *r*_1_ is the center radius of the Fresnel-like pattern and *λ* is the wavelength of the probe beam. Herein, the focal length of the proposed Fresnel lens is about 39.5 cm.

## 3. Results and Discussion

### 3.1. Recording Intensity and Concentration Study

**Figure 2:**
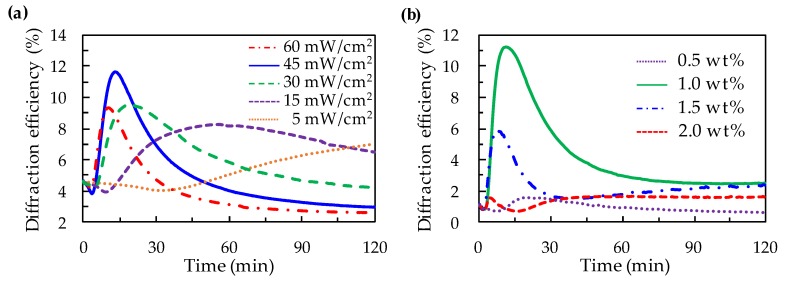
(**a**) The diffraction efficiency of a 1.0 wt% 12 μm ADDLC sample as a function of time irradiated by the green beam with various intensities; and (**b**) the diffraction efficiency of a 12 μm ADDLC sample with various doping concentrations of methyl red (MR) as a function of time irradiated by a green beam with the intensity of 45 mW/cm^2^.

The following two cases are discussed first in order to find the optimal experimental parameters for fabricating the LC Fresnel lens. (1) Fixing the doping concentration of MR in the ADDLC sample, the diffraction efficiency as a function of the irradiated time is measured with various intensities of the green beam. (2) At a specific intensity of the green beam irradiation, the diffraction efficiency modulated with the irradiated time is detected in the samples with various doping concentrations of MR. Figure 2a displays the diffraction efficiency of the 1.0 wt% ADDLC sample as a function of time with various intensities (5–60 mW/cm^2^) of the green beam. The diffraction efficiency is defined as the focusing intensity of the sample divided by the *I*_0_, which is the intensity of the incident light. The results show that the time of the sample to reach the maximum diffraction efficiency decreases as the irradiated intensity of the green beam increases. The focusing effect of the sample is induced by the difference in the refractive indexes between the odd and even zones. When the irradiated intensity of the green beam increases, more azo dye molecules go through *trans*–*cis* photoisomerization. Based on the guest–host effect between the molecules of LCs and MR, the alignment of the LCs in the odd zones reorientation into the TN structure will be much easier as the irradiated intensity increases. However, when the irradiated intensity of the green beam is 60 mW/cm^2^, the excessive dye molecules undergoing *trans*–*cis* photoisomerization will cause a slight disorder of the structure of TN-LCs at the surface of the control substrate, resulting in the decrease of the maximum diffraction efficiency. The diffraction efficiency has the highest value when the irradiated intensity of the green beam is 45 mW/cm^2^, and the time it takes to reach the maximum diffraction efficiency is only 12 min. As the exposure time continues to increase, the dye molecules in the even zones start to go through *trans*–*cis* photoisomerization and the LCs reorientation into TN structure also occurs in the even zones, which leads to a gradual decrease in the phase difference between the odd and even zones. The diffraction efficiency gradually declines when the exposure time is above 12 min.

Figure 2b presents the diffraction efficiency of the ADDLC samples with various doping concentrations (0.5–2.0 wt%) of MR modulated with the time irradiated by the green beam with an intensity of 45 mW/cm^2^. The time for the sample to reach the maximum diffraction efficiency decreases when the doping concentrations of MR increase. The experimental result indicates that more doping concentrations of MR cause the LCs in the odd zones to reorient into TN structures more easily. The diffraction efficiency has its highest value when the doping concentration of MR is 1.0 wt%, and the time to reach the maximum diffraction efficiency takes only 12 min. From the results shown in Figure 2a,b, the optimal parameters for fabricating the LC Fresnel lens can be summarized as those for an ADDLC sample with a doping concentration of 1.0 wt%, irradiated by a green beam with the intensity of 45 mW/cm^2^ for 12 min.

**Figure 3:**
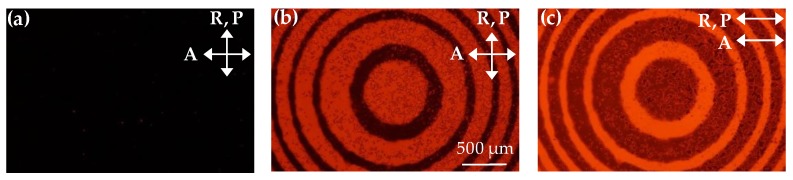
The polarized optical microscope (POM) images of a 1.0 wt% ADDLC sample irradiated by a green beam with intensity of 45 mW/cm^2^ for: (**a**) 0 min and (**b**) 12 min under P⊥A, and (**c**) the same irradiated conditions under P//A. P and A are the directions of the polarizer and analyzer in the microscope, respectively, and R is the alignment direction of the sample.

The 1.0 wt% ADDLC sample irradiated by the green beam with the intensity of 45 mW/cm^2^ for 0 and 12 min is observed via a polarized optical microscope (POM), as shown in Figure 3. When there is no green beam irradiating on the sample, the direction of the LC molecules is aligned along the rubbing direction homogeneously, resulting in the dark state under the POM with crossed polarizers (P⊥A), as shown in Figure 3a. As the irradiation time of the green beam increases, the molecules of the MR in the bright (odd) zones of the Fresnel-like pattern go through a *trans*–*cis* photoisomerization reaction and lead to an original homogeneous-alignment LC reorientation into the TN structure. Based on the polarization rotation effect of the TN-LCs, the brightness in the odd zones will gradually increase while the even zones will maintain the dark state under a cross polarized microscope. Figure 3b,c shows the POM images of the sample irradiated by the green beam for 12 min observed under crossed polarizers and parallel polarizers, respectively. The optical behaviors of the adjacent zones are complementary to each other.

### 3.2. AC Voltage Dependence

**Figure 4:**
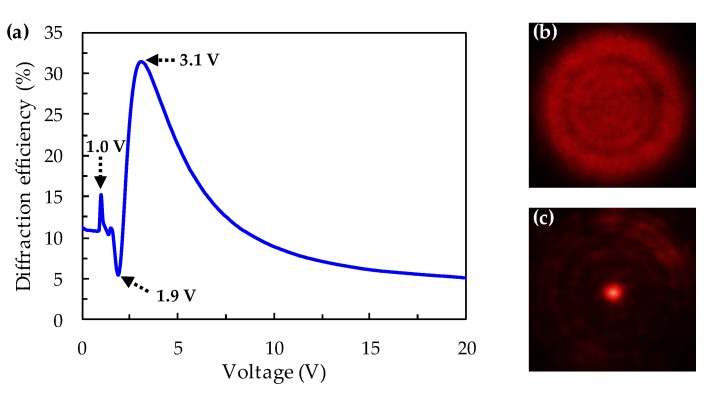
(**a**) The diffraction efficiency of the Fresnel lens depends on the applied alternating-currents (AC) voltage under the conditions of 1.0 wt% MR doping and the illumination of green beam with intensity of 45 mW/cm^2^ for 12 min. The features at particular voltages 1.0 V, 1.9 V, and 3.1 V have been labeled. (**b**) The optical images on the focal plane observed by using a charge-coupled device (CCD) camera at the initial state of the sample. (**c**) The optical images on the focal plane when the sample is applied with an AC voltage of 3.1 V.

The experiment further investigates the diffraction efficiency of the LC Fresnel lens as a function of the applied AC voltage, as shown in Figure 4a. The focus ability of the LC Fresnel lens is mainly contributed to by the difference of the refractive indices between the odd and even zones. The phase difference (Γ) of the adjacent zones can be written as:

(2) $$\Gamma =\frac{2\pi d(n_{odd}-n_{even})}{\lambda },$$

where *d* is the thickness of the ADDLC film, *n_odd_* and *n_even_* represent the effective refractive indices of the odd and even zones, respectively, and *λ* is the wavelength of the incident light. When the phase difference between the adjacent zones is approximately equal to *π*, the diffraction (focusing) efficiency of the LC Fresnel lens is at its maximum at the main focus (first order) of [23,24]:

(3) $$\eta_{1}={\left(\frac{\sin(\frac{\pi }{2})}{\frac{\pi }{2}}\right)}^{2}\approx 41\%.$$

Figure 4a shows that when the applied voltage is 0 V, the proposed Fresnel lens has a slight diffraction efficiency of about 11% due to the difference in the liquid crystal structure between the odd and even zones. When the applied voltage is below 0.8 V, the diffraction efficiency of the Fresnel lens scarcely changes because the applied voltage is lower than the threshold voltage of the liquid crystals. A significant change of the diffraction efficiency occurs when the applied voltage is above 0.8 V. The molecules of TN-LCs are caused to start to reorient by the external electric field, resulting in an increasing phase difference between the odd and even zones. There is a small peak of 15% when the applied voltage is 1.0 V, as shown in Figure 4a. One possible reason is that the refractive index changes in the center bulk of the liquid crystal film, which is affected by the weaker alignment anchoring force. When the applied voltage reaches 1.9 V, the diffraction efficiency has a minimum of about 5% because the phase difference between the adjacent zones equals approximately 2*π*, indicating that the Fresnel lens now is at an unfocused state. As the applied voltage gradually increases to 3.1 V, the diffraction efficiency of the Fresnel lens reaches its maximum of 31.5%; herein the phase difference between the adjacent zones is equal to *π*. Finally, the LC molecules in the adjacent zones are gradually turned parallel to the direction of the electrical field when the applied voltage’s continuously increasing results in the diffraction efficiency slowly decrease to the minimum value (5%). The optical images of the 1.0 wt% ADDLC sample on the focal plane captured by a charge-coupled device (CCD) camera are shown in Figure 4b,c. When there is no green beam irradiation and no AC voltage, the focusing effect of the sample does not occur and the spot of the probe beam exhibits a uniform distribution on the focal plane, as shown in Figure 4b. The interference ring in the beam spot is mainly caused by the aperture (iris) in front of the sample, which does not affect the focusing effect of the sample or the measurement of the experiment. When the sample is irradiated by the green beam with an intensity of 45 mW/cm^2^ for 12 min and then an AC voltage of 3.1 V is applied, the diffraction efficiency of the sample reaches the maximum value. Figure 4c presents a far field image when the sample is operated for focusing light. The red beam propagates through the sample and is focused on the focal plane. Therefore, the red light in the effective area of the Fresnel-like pattern is concentrated at the center in focus and leads to the bright focal point observed in Figure 4c.

**Figure 5:**
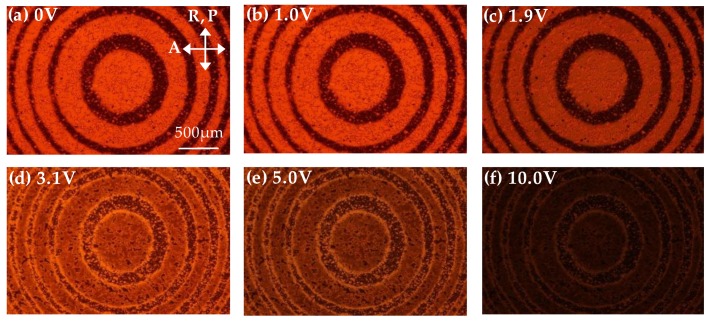
The POM images of the Fresnel-like pattern in the ADDLC sample applied by various AC voltages of: (**a**) 0 V; (**b**) 1.0 V; (**c**) 1.9 V; (**d**) 3.1 V; (**e**) 5.0 V; and (**f**) 10.0 V, respectively. P and A are the directions of the polarizer and analyzer in the microscope, respectively, and R is the alignment direction of the sample.

The POM images of the Fresnel-like patterned ADDLC sample operated with various AC voltages are shown in Figure 5. The POM images of the Fresnel-like pattern in Figure 5a–f were taken at the applied voltages of 0, 1.0, 1.9, 3.1, 5.0, and 10.0 V, respectively. In Figure 5b, the Fresnel-like pattern barely changes even though the applied voltage exceeds the threshold voltage of the LCs. A possible reason is that the applied voltage has not reached the optical threshold voltage of TN-LCs. When the applied voltage is 1.9 V, the optical intensity in the odd zones gradually decreases since the molecules of the TN-LCs are reoriented by the electrical field, as shown in Figure 5c. In the even zones of the Fresnel-like pattern, the orientated direction of the homogeneous-alignment LCs is parallel to the transmission axis of the polarizer in the microscope, resulting in a small optical change. When the applied voltage continues to increase, the molecules of the TN-LCs are induced by the increased voltage and gradually turned parallel to the direction of the electrical field. The polarization rotation effect of the TN-LCs begins to disappear, resulting in a gradual decrease of the optical intensity in the odd zones, as shown in Figure 5d–f. It is worth noting that bright rings can be observed at the boundary of the odd and even zones. The leakage of light results from the discontinuous orientation at the boundaries of the adjacent zones since the arrangement of LC molecules exhibits short range continuity.

### 3.3. Polarization Dependence

**Figure 6:**
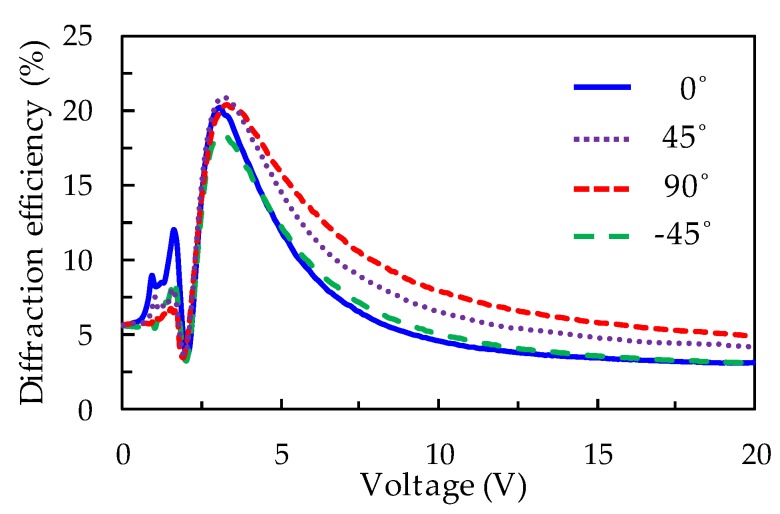
The variations of diffraction efficiency of the Fresnel lens on the applied AC voltage probed by various linearly polarized beams.

In order to investigate the polarization dependence of the LC Fresnel lens, the diffraction efficiency of the Fresnel lens probed with various linearly polarized beams is measured as a function of voltage, as shown in Figure 6. The angles between the various directions of the linearly polarized red beam and the *x*-axis defined in Figure 1 are adjusted as 0°, ± 45°, and 90°, respectively. The experimental results show that at a low voltage (<1.9 V), the Fresnel lens has a significant polarization dependence on the incident red beam. This is probably because the polarization rotation effect is caused by the TN-LCs when the red beam incidents on the odd zones. The diffraction efficiency of the Fresnel lens is contributed to by the phase difference and the superposition of light polarization between the adjacent zones. When the polarized direction of the red beam is parallel to the *x*-axis (0°), the diffraction efficiency of the Fresnel lens is clearly observed, whereas the diffraction efficiency is nearly imperceptible when the polarized direction of the red beam is vertical to the *x*-axis (90°). Furthermore, when the applied voltage is above 1.9 V, the polarization rotation effect disappears because the molecules of TN-LCs in the odd zones are gradually turned parallel to the direction of the electrical field. Herein, the diffraction efficiency is only contributed to by the phase difference between the adjacent zones. At the high applied voltage region, the phase differences between the adjacent zones are nearly equal for the various directions of the linearly polarized red beam. Therefore, the LC Fresnel lens shows the polarization independence.

### 3.4. Rewriting

**Figure 7:**
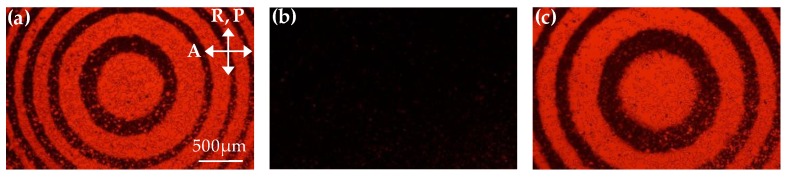
The POM images for rewriting different Fresnel-like patterns in the Fresnel lens: (**a**) A Fresnel-like pattern is formed under 1.0 wt% MR doping and writing intensity of 45 mW/cm^2^ for 12 min; (**b**) the pattern is erased through a red beam with 1.04 mW/cm^2^ exposure and heating at 120 °C for 5 min; (**c**) a new, different pattern is rewritten by using the same experimental conditions as (a). P and A are the directions of the polarizer and analyzer in the microscope, respectively, and R is the alignment direction of the sample.

The rewriting characteristic of the LC Fresnel lens is investigated and introduced in the final part of this letter. First, the 1.0 wt% MR doped TN-LC sample is irradiated by the green beam with an intensity of 45 mW/cm^2^ for 12 min. The Fresnel-like pattern is lithographed on the sample and can be observed by the cross polarized microscope, as shown in Figure 7a. In the odd zones of the Fresnel-like pattern, the alignment of the liquid crystals turns into a TN structure because the azo dyes of MR are exposed by the green beam, whereas, the liquid crystals are maintained in a homogeneous-alignment structure in the even zones. Then, the recorded Fresnel-like pattern is erased by heating at 120 °C. In order to shorten the removal time of the pattern, a red beam with an intensity of 1.04 mW/cm^2^ is additionally employed to irradiate on the sample due to the slight absorption of the *cis*-isomer of MR at 632.8 nm. Figure 7b shows the POM image of the sample after heating and exposing with a red beam for 5 min. The initial Fresnel-like pattern is completely erased. The dark state of the POM image indicates that the liquid crystal molecules in the sample have been reoriented into a homogeneous alignment, which proves that the photoalignment effect of the MR can be erased. Finally, a new Fresnel-like pattern with a different size is rewritten in the same position of the sample with identical parameters, as shown in Figure 7c. The larger Fresnel-like pattern presented in Figure 7c demonstrates that the proposed Fresnel lens can be erased and rewritten. With this capability, Fresnel lenses with various focal lengths can be fabricated by irradiating the Fresnel-like pattern of the green beam with different sizes on the ADDLC sample.

## 4. Conclusions

A diffraction efficiency and focal length tunable Fresnel lens with TN-LCs has been proposed in this letter. The photo-alignment effect of the azo dyes is produced in the odd zones when the green beam forming the Fresnel pattern from a Sagnac interferometer is irradiated on the sample. The Fresnel lens is generated with the phase difference of the various structures of liquid crystals in the odd and even zones. The experiment first investigates the focusing feature of the Fresnel lens with various concentrations of doped MR and various intensities of the green beam. The optimal focusing feature of the Fresnel lens occurs when the MR concentration is 1.0 wt%, and the illumination of the green beam is set as 45 mW/cm^2^ for 12 min. The diffraction efficiency of the Fresnel lens can be electrically controlled to its maximum value of 31.5% under the applied voltage of 3.1 V. Furthermore, the diffraction efficiency of the Fresnel lens shows the significant polarization dependence at a low applied voltage (<1.9 V), but the polarization independence when applying voltage is above 1.9 V. In addition, the photo-alignment effect of the azo dyes can be erased thermally and rewritten with a different Fresnel-like pattern, which leads to the possibility of fabricating a focal length tunable Fresnel lens.
